# Analytical chemistry in front of the curtain!

**DOI:** 10.1007/s00216-024-05128-9

**Published:** 2024-01-24

**Authors:** Erwin Rosenberg, Rudolf Krska

**Affiliations:** 1 Institute of Chemical Technologies and Analytics, Vienna University of Technology, Getreidemarkt 9/164-AC, 1060 Vienna, Austria; 2 University of Natural Resources and Life Sciences, Vienna (BOKU), Department of Agrobiotechnology IFA-Tulln, Institute of Bioanalytics and Agro-Metabolomics, Konrad-Lorenz-Str. 20, 3430 Tulln, Austria; 3 Institute for Global Food Security, School of Biological Sciences, Queens University Belfast, University Road, Belfast, Northern Ireland BT7 1NN UK; 4 Austrian Competence Centre for Feed and Food Quality, Safety and Innovation FFoQSI GmbH, Konrad-Lorenz-Str. 20, 3430 Tulln, Austria

**Keywords:** Evolution of analytical chemistry, Societal impact, Education, Measurement science, Image of analytical chemistry, Scientific metrics

## Abstract

This feature article discusses the enabling role of analytical chemistry in important fields of research and development such as life science, material sciences and environmental sciences. It comments on the often limited visibility of analytical sciences in the public perception and suggests ways to overcome this shortcoming and to create bigger impact.

**Graphical Abstract:**

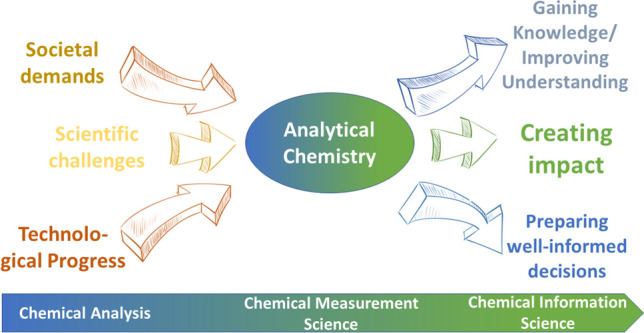

## Introduction

Analytical chemistry has continuously evolved and made enormous progress particularly in the years after the turn of the millennium: The developments in all areas of analytical chemistry — surface analysis, materials analysis, sensors, hyperspectral imaging and micro- and nanodomain analysis or bioanalysis, to name just some few ‘buzzwords’ of the past years — have led to significant progress in the various related disciplines. Although perhaps not always seen and appreciated in this function, analytical chemistry clearly has become the ‘enabling science’ to many areas of chemistry and to other scientific fields such as life sciences, medicine, environmental science or materials science. However, this awareness is hardly present in the general public, and the contribution of analytical chemists is often perceived as a routine service rather than a scientific one. We want to discuss in this feature article why we believe this is so (and what can be done to overcome this).

## The past

Analytical chemistry has a long tradition in German-speaking countries. It is not just a coincidence that the oldest journal in the field of analytical chemistry, the *Zeitschrift für Analytische Chemie* — the predecessor of the current journal *Analytical and Bioanalytical Chemistry* — was founded in 1861 by the eminent German scientist Carl Remigius Fresenius after realising at the *Karlsruhe Congress* (organised during September 3–5, 1860, in Karlsruhe on the initiative of Kekulé, Wurtz and Weltzien and representing the first international symposium of modern chemistry [1]) how different the views on chemistry were [2].

In a *prospectus* that Fresenius wrote to announce the new journal that he had decided to edit, he stated that ‘without doubt it can be demonstrated that all major advancements in chemistry more or less directly depend on the availability of new and improved analytical methods […]’ [3]. Clearly, Fresenius at that time already envisaged the role of analytical chemistry as an enabling science, rather than merely a tool to be used by other chemical disciplines. Although this view was the credo and understanding of many generations of analytical chemists after Fresenius [4], in reality, analytical chemistry was usually not seen as an own discipline but as part of inorganic, organic or biochemistry. The (sometimes historic) names of many departments (‘institutes’) of German-speaking universities are indicators for this situation where often analytical chemistry formed part of larger departments, such as an ‘Institute of Inorganic *and* Analytical Chemistry’ or ‘Institute of Organic *and* Analytical Chemistry’), with analytical chemistry usually being added as an appendix to another scientific discipline. It appears that analytical chemistry has to stronger emphasise both its importance among and its independence from the other fields of chemistry. The hard facts speak a clear language: It is quite a remarkable fraction of the Nobel Prizes in Chemistry that were awarded to analytical chemists or chemists that have developed analytical methods (Table 1) — among these, the 1993 Nobel Prize to Kary B. Mullis for his invention of the polymerase chain reaction (PCR) method which has become so important recently in the course of the current COVID-19 pandemic — although there are typically the Nobel Prizes awarded to physical chemists and synthetic (organic) chemists that capture the attention (and apparently also more intensively stimulate the imagination) of the general public.

**Table 1:** Important analytical developments awarded with the Nobel Prize in Chemistry [5]

Name	Achievement	Year awarded
Francis William Aston	‘for his discovery, by means of his mass spectrograph, of isotopes, in a large number of non-radioactive elements, and for his enunciation of the whole-number rule’	1922
Fritz Pregl	‘for his invention of the method of micro-analysis of organic substances’	1923
George de Hevesy	‘for his work on the use of isotopes as tracers in the study of chemical processes’	1943
Arne Wilhelm Kaurin Tiselius	‘for his research on electrophoresis and adsorption analysis, especially for his discoveries concerning the complex nature of the serum proteins’	1948
Archer John Porter Martin and Richard Laurence Millington Synge	‘for their invention of partition chromatography’	1952
Jaroslav Heyrovsky	‘for his discovery and development of the polarographic methods of analysis’	1959
Sir Aaron Klug	‘for his development of crystallographic electron microscopy and his structural elucidation of biologically important nucleic acid-protein complexes’	1982
Richard R. Ernst	‘for his contributions to the development of the methodology of high-resolution nuclear magnetic resonance (NMR) spectroscopy’	1991
Kary B. Mullis (1/2, shared with Michael Smith, 1/2)	‘for his invention of the polymerase chain reaction (PCR) method’ (K.B.M.) and ‘﻿for his fundamental contributions to the establishment of oligonucleotide-based, site-directed mutagenesis and its development for protein studies’ (M.S.)	1993
John Fenn and Koichi Tanaka (each 1/4) and Kurt Wüthrich (1/2)	‘for their development of soft desorption ionisation methods for mass spectrometric analyses of biological macromolecules’ (J.F., K.T.) and ‘for his development of nuclear magnetic resonance spectroscopy for determining the three-dimensional structure of biological macromolecules in solution’ (K.W.)	2003
Eric Betzig, Stefan W. Hell and William E. Moerner	‘for the development of super-resolved fluorescence microscopy’	2014
Jacques Dubochet, Joachim Frank and Richard Henderson	‘for developing cryo-electron microscopy for the high-resolution structure determination of biomolecules in solution’	2017

## The present

Since the development of modern analytical chemistry [6], the role of chemical analysis has changed significantly, and its focus has broadened enormously. While many ground-breaking developments in the field of analytical chemistry, such as the highly sensitive tandem mass spectrometric methods and ‘omics’ technologies, are more likely to be attributed to the physicists and bioinformatics, analytical chemistry has become indispensable as an ‘enabling discipline of science’ for answering interdisciplinary questions [7]. The data produced by analytical chemists or with analytical methods serve as an essential basis for decision-making in many areas of research and everyday life. This is especially true for the life sciences, but also for materials research and food safety and for recording the state of the environment and long-term effects of climate change. In addition to targeted analysis, non-directional ‘omics’ approaches for the holistic evaluation of biological systems through their transcriptome, proteome or metabolome have become indispensable as the basis for much of our understanding of the functioning, interaction and communication of biological systems. Using modern metabolomics-based methods for studying the interactions between microbes and plants, analytical chemists help answer fundamental biological questions. This is particularly true for the life sciences, but also for the safety of the food chain, for smart farming or for monitoring the long-term effects of climate change or the introduction of man-made chemicals in the environment — all of these fields of application critically rely on analytical methods such as multi-class analytical methods [8] where often by the use of hyphenated techniques, hundreds of agrochemicals [9], metabolites [10] or environmental contaminants [11] can be determined simultaneously in one run. Moreover, modern hyphenated techniques such as high- and ultra-high performance liquid chromatography coupled with high-resolution time-of-flight mass spectrometry (UHPLC/TOF–MS) are capable of producing an enormously large, in the best case even comprehensive amount of data on the sample investigated. This may be considered as an ‘electronic archive’ [12] which retains all the information on one given sample, even if the sample itself is no longer physically existing. The comprehensive recording of mass spectral data with a TOF–MS instrument or an Orbitrap mass spectrometer [13] would thus allow going back to recorded data to verify, for example, if a particular compound had already been present in a given sample, even if it had gone undiscovered at the time of analysis. The possibility to interrogate MS data at a later point in time would also be of great advantage if the influence of climate change on the (co-)occurrence of secondary metabolites of fungi, plants and bacteria is to be studied over a long period of time. The analytical chemist is more than ever required to create a data base for the occurrence and behaviour of contaminants and other relevant substances in foods and the environment and to act as a link between the most diverse scientific disciplines.

In their excellent *feature paper* in this journal [4], Adams and Adriaens have described this change in the philosophy of analytical chemistry as a ‘metamorphosis’, a paradigm change. The key aspects of this paradigm change identified by those authors are that analytical chemistry has moved:

- from simple measurements to combinations of tools and techniques producing and capable of handling ‘big’, multi-parametric data (multispectral or hyperspectral data, multiplexing of instrumental approaches, processing of many samples, etc.),
- from problem-driven applications to discovery-driven (hypothesis generating) use of analytical tools,
- to addressing increasingly complex issues for studying nature and materials,
- to adopting a systemic (holistic) approach rather than one based on unit operations, based on individual measurements.

When we criticise that the fundamental and bridging role of analytical chemistry often remains invisible or is not optimally received [14, 15], then this is because either we ourselves or our colleagues from other disciplines have not yet made this paradigm shift and still stay with some more conventional definition and understanding of the role and scope of analytical chemistry. In an even earlier feature article in this journal, the late Miguel Valcárcel contested the (unfavourable) image of analytical chemistry in the public and among peers which he assigned to both the lack of self-esteem of analytical chemists and (at least partly) due to the lack of understanding and awareness of representatives from other scientific disciplines, who often have little imagination of the complexity of the analysis, the required rigidity in the methodological approach to produce valid and high-quality data and the limitations of the interpretability of the results. Worse still, the services of analytical chemists are often perceived as routine work that increasingly takes place in service units — the so-called core facilities which on the upside provide analytical services needed to support research projects in other scientific fields. On the downside, this development potentially undermines the position of institutes dedicated to research in analytical chemistry. This clearly calls for better communication of our role and for better education of the coming generations of chemists and other scientists and for measures to increase the awareness for the importance of analytical chemistry as scientific discipline.

Although international funding agencies, such as the European Commission, have provided grants for innovative research projects linked with chemical analysis or analytical methods development in the past, funding agencies do usually not feel responsible for and are rarely prepared to fund the development of fully validated analytical methods, even though these represent the basis for many studies. Even essential research work on the extensive evaluation of extraction protocols, such as those required in the metabolomics arena, is often viewed as uncreative or not enough visionary. On the other hand, there is substantial public funding going to ASTM and CEN to mention two of the largest standardisation bodies. In the EU, CEN receives its budget from DG GROW and specific policy DGs in the European Commission; e.g. DG Environment funded the development of standard methods, e.g. for priority substances in waters including their validation (mandate 424 to TC230). In that sense, it is correct that validation of analytical methods per se is not routinely supported, but on the other hand funding can still be secured for such studies through DG RTD.

Clearly, it is of course also the task of analytical chemists and their professional societies to face the beforementioned challenges more than ever and to make their work more visible — especially outside of their own community, and even more so in the public. Fellow scientists as well as laymen must become more aware of the central and enabling role that analytical chemistry has in providing the data and preparing decisions of far-reaching societal relevance but also in enabling progress in almost any other scientific discipline. Surprisingly, this is not easy to achieve, as many of the analytical methods in routine use have reached such a high level of reliability and perfection that they provide a 24/7 availability on a ‘push-button’ basis. Nowadays, analytical instrumentation is designed to work reliably, be robust, have maximum uptime, high selectivity and sensitivity and be usable with a large range of different samples. This often leads to a ‘black box’ design where the user is hardly aware of the hidden complexity and the ingenious design of the instrumentation and the analytical protocol that lead to the desired result — see, for example, mass spectrometric detectors for liquid chromatography which are advertised by the manufacturer as providing the possibility of ‘routinely generating high-quality mass spectra without the need for any special training or expertise’ [16]. It appears that such developments take account of the fact that analytical chemistry is the by far most often named job description of European chemistry graduates, while only a much smaller fraction of these graduated with this specialisation [17]. In view of the above said, such philosophy must also be criticised as being contra-productive to the declared aims as it downsizes the important role of trained staff in analytical chemistry.

## Creating impact

The field of application where analytical chemistry has made the greatest impact is, without doubt, that of the biosciences. To create a deeper understanding of the role of the individual components in biological systems — such as proteins, lipids, DNA, metal ions and many more — and their formation, metabolisation and physiological relevance, analytical chemistry is essential. Many of the great discoveries in biosciences have been driven by the availability of appropriate analytical techniques, and this still continues to be so. The age of the ‘omics’ technologies (proteomics, metabolomics, genomics) would not have come without the heavy involvement of modern analytical techniques and instrumentation. For instance, genomics relies heavily on DNA microarray technology, proteomics on mass spectrometry and metabolomics on hyphenated gas and liquid chromatographic techniques.

The field of biosciences is thus an excellent example for how analytical chemistry contributes to the development of this scientific area by the development of new technology, new methods and even new reagents.

All of these are important and are the result of the out-of-the-box thinking of creative minds who were and are not willing to accept the limitation of the technology of their times but provided new approaches to solve their problems, very often based on a multi- or interdisciplinary background. When Tswett was looking for a possibility to separate plant chlorophylls, he invented chromatography [18], and in order to demonstrate the existence and to quantify the relative composition of different isotopes of various elements, Aston developed what later was considered the first working mass spectrometer [19] (and was, in contrast to Tswett, awarded the Nobel Prize in Chemistry in 1922 for his ground-breaking invention; see also Table 1). Both techniques are nowadays, more than a century after their invention, indispensable in the analytical laboratory and have shaped modern natural sciences and enabled many of the important discoveries. It should be mentioned, however, that the development of chromatography as an analytical technique was (with the Nobel Prize going in 1952 to Martin and Synge) later duly recognised. At this point, the question could be asked, whether science is mostly driven by ideas (that require the development of appropriate tools) or rather by tools (such as new analytical methodologies and instrumentation) that would allow investigating new ideas [20]. We will, however, not start this chicken-or-egg discussion here. Instead, we reiterate that the best foundation to achieve recognition in science is to have impact. This brings us to redefine the term ‘impact’: Impact as it is meant in this context is not impact factor (of a journal) or Hirsch-(*h*-)factor of an individual researcher — as a side note, you may find it worthwhile knowing that the *h*-factor of Albert Einstein who certainly is one of the ‘icons’ of modern science and one of its most important representatives is ‘only’ 43 [21], a value which is reached and even surpassed by many accomplished scientists of our times, including one of the authors of this paper. Instead, impact shall be defined here as real-life impact or practical usability. This can be judged by how the analytical tools and protocols developed by analytical chemists are picked up by other scientists. A very good indicator for real-life applicability is the list of the ten most quoted publications in science (Table 2): It may be surprising to see that not the most important theoretical findings such as Einstein’s theory of general relativity can be found at the top of this list, but a very pragmatic paper that describes a novel method for the quantitative determination of proteins by the Folin reagent [22]. Altogether, the top ten list of the most quoted papers in science is dominated by seven papers that describe analytical techniques (and reagents) in the biochemical laboratory and one bioinformatics technique that have become the basis for many important developments and discoveries particularly in biosciences. The other papers, all of them having received well over 50,000 citations, are from physical chemistry (2) and crystallography (1).

**Table 2:** The 10 most cited articles in chemistry

Rank	Citations^1^	Title	Subject	Year	Author(s)	Reference
1	351,269	Protein measurement with the Folin phenol reagent	Biology lab technique	1951	Lowry OH, Rosebrough NJ, Farr AL & Randall RJ	J Biol Chem (1951) 193: 265–27
2	256,211	Cleavage of structural proteins during the assembly of the head of bacteriophage T4	Biology lab technique	1970	Laemmli UK	Nature ‏(1970) 227: ‏680–685
3	233,152	A rapid and sensitive method for the quantitation of microgram quantities of protein utilising the principle of protein–dye binding	Biology lab technique	1976	Bradford MM	Anal Biochem (1976) 72: 248–254 ‏
4	86,210	Development of the Colle-Salvetti correlation–energy formula into a functional of the electron density	Physical chemistry	1988	Lee C, Yang W, Parr R	Phys Rev B (1988) 37:‏ 785–789
5	76,325	A short history of SHELX	Crystallography	2008	Sheldrick GM	Acta Crystallogr A (2008) 64: 112–122
6	72,173	Density-functional thermochemistry. 3. The role of exact exchange	Physical chemistry	1993	Becke AD	J Chem Phys (1993) 98: 5648–5652
7	70,057	Basic local alignment search tool	Bioinformatics	1990	Altschul SF, Gish W, Miller W, Myers EW & Lipman DJ	J Mol Biol (1990) 215: 403–410
8	69,361	DNA sequencing with chain-terminating inhibitors	Biology lab technique	1977	Sanger F, Nicklen S, Coulson AR	Proc Natl Acad Sci USA (1977) 74: 5463–5467
9	66,822	Single-step method of RNA isolation by acid guanidinium thiocyanate-phenol–chloroform extraction	Biology lab technique	1987	Chomczynski P, Sacchi N	Anal. Biochem. (1987) 162: 156–159
10	61,048	A simple method for the isolation and purification of total lipides from animal tissues	Biology lab technique	1957	Folch J, Lees M, Stanley G	J Biol Chem (1957) 226: 497–509

^1^As of 04/08/2023, retrieved from Web of Science, Clarivate Analytics

**Fig. 1:**
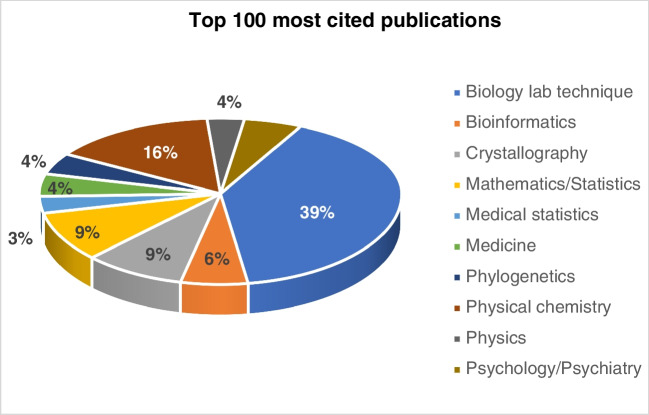
Top 100 most cited publications according to the scientific discipline. Biology lab techniques dominate the top 100 list, followed by papers in physical chemistry and crystallography [26]

**Fig. 2:**
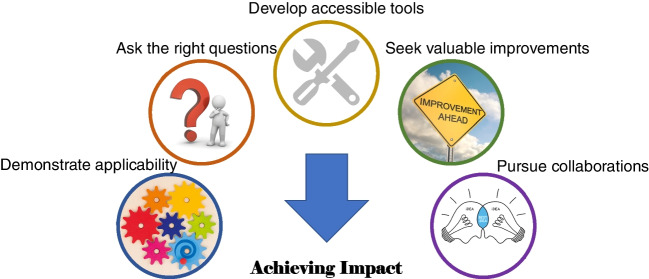
How to achieve impact with analytical work (inspired by Hare and New [23])

While these numbers already speak a clear language, they still do not provide a satisfactory answer to the question why recognition for the field of analytical chemistry may yet not be as widespread. A possible explanation is that many achievements and papers of analytical chemists are not identified as genuine analytical work but are attributed to the field of science in which analytical chemistry has been applied (Fig. 1). Hence, it is more likely that highly recognised colleagues find their names, e.g. among the top ranked toxicologists or food safety experts rather than in one of the few rankings dedicated to analytical chemistry. Thus, whilst we clearly have to raise awareness for our scientific field, we shall also consider a number of other aspects that we believe are of importance to maximise impact and awareness of analytical chemistry (Fig. 2) [23].

### Choose the right research topic

Be considerate when choosing the subject of your work: Of course, we love what we do, and the best pre-condition for being good and successful at what you do is the full commitment and dedication to this work. However, it will very much depend on the choice of research topic, that is, whether it is one of the relevant research questions that you address in your work and consequently your work will find public attention or rather remain unnoticed.

### Develop accessible tools

A key component of the work of analytical chemists is the development of new analytical tools. The word ‘tools’ shall mean in this context both instrumentation, reagents and protocols. This development may either be directed at ‘making measurable what could not be measured before’ (in the spirit of Galileo Galilei), but also at simplifying existing protocols or methods to make them more accessible and widely accepted by the scientific and professional community. Both are perfectly valid motivations for scientific research in analytical chemistry, and two examples for the latter are the introduction of solid-phase microextraction (SPME) by Pawliszyn and Arthur in 1990 [24] with its continuously increasing popularity or that of the QuECHERS procedure for the sample preparation of pesticides in food and other samples by Anastassiades, Lehotay and colleagues [25] in 2003. Due to their operational simplicity, versatility and adaptability, both methods have very quickly reached great popularity and have been used in a great number of studies since.

### Demonstrate the potential impact of analytical tools

Particularly those working in the field of biomedical and pharmaceutical analysis know very well the inertia of these fields to adopt new analytical tools and methodologies. The enormous administrative and lab work-related effort to replace an established methodology in a laboratory working in a regulated environment by a new one is an enormous obstacle for the early and easy adaption of a new technology. Unless it is proven — preferably by those who have developed this new technology, protocol or instrument — that the novel procedure performs significantly better than the previous one, it will not be introduced in practice. Only if analytical chemists have demonstrated, through thorough validation of the newly proposed protocol or technology that this surpasses the established one and that it provides a significant gain in analytical information, robustness, reliability, sample throughput or simply cost savings, there is a chance for a wider acceptance of their new developments.

### Pursue collaborations

As simple as this sounds, it is essential for our ideas to be tested in practice and to search for collaborators and collaborations where the analytical tools that were developed prove to be useful. The already existing intensive collaboration of analytical chemists and scientists from other fields of application can be further fertilising for both sides, both pointing to the need of the new tools to be developed and demonstrating their ability to answer new research questions.

### Academic impact

In the attempt to make scientific quality measurable [27], scientific impact — in its original sense of breadth or depth of effect — is increasingly replaced by ‘impact factor’. Unfortunately, the impact factor of a journal is often (and incorrectly) considered as an indicator for the quality and the importance of a manuscript that was published in this journal, which not necessarily is the case [28]. For this reason, a number of alternative measures of scholarly impact have been evaluated over the years and are eventually also adopted by funding organisations [29]. Nonetheless, the demonstration of impact in the scientific literature by publishing in journals with high-impact factors is the most obvious route for securing funding for future projects.

Ironically, analytical chemists tend to maximise their impact by developing, improving or modifying their analytical tools which gives them the chance to publish their work (in high-impact journals), rather than to search for collaborations and demonstrate that the tools they have developed are really useful for the scientific community on large scale. Academic and practical impact may thus be rivals rather than friends, as analytical chemists may be forced to prioritise academic over practical impact to secure funding in a competitive environment. Instead of demonstrating the true impact of a newly developed analytical tool, analytical chemists must rather strive to demonstrate potential impact to the funding bodies. In this context, it is worthwhile mentioning that the top three papers in the list of the most cited articles, all three of them having received more than 100,000 citations (Table 2), are all analytical techniques for the biochemical laboratory. This demonstrates that academic impact can, after some time, also lead to practical impact.

### Practical impact

All of what was said before strives at achieving impact in practice. Following one, or better, several of the above strategies may prevent that the newly developed tool is not taken up by the scientific community or not used beyond its first report. One of the best strategies for achieving immediate practical impact is the close collaboration of analytical scientists with those who seek to benefit from their developments. When this is the case, not only does the analytical scientists have direct access to the stakeholders they target for future applications, but these stakeholders can also directly communicate the needs and critical issues that may not immediately be apparent to the analytical scientist. Two prominent examples for this statement are the development of gas chromatography/mass spectrometry (GC/MS) and that of the CRISPR gene editing technique. The former technique had not been invented at a university lab, but by McLafferty and Gohlke at the Midland, MI, laboratories of Dow Chemicals where the two scientists developed this technique which later became the workhorse of organic analytical chemistry in response to the need of their lab colleagues to characterise individual substances in a mixture [30]. The second example, the gene editing technique CRISPR whose huge potential in biology, medicine and life science was recognised through the award of the Nobel Prize in Chemistry to Doudna and Charpentier, would not have been possible without the ability to measure and detect the outcome of the ‘chemical reprogramming’ of a gene. However, it was not a single analytical advance that made CRISPR possible, but instead decades of development and refinement of biochemical analytical techniques in constant interaction with the users of this technique were necessary to develop those (bio)analytical tools that eventually led to the discovery of these revolutionary ‘genetic scissors’ [31].

## The future

So, what are the lessons learnt from the current situation ? What can be done to improve the current situation and to help analytical science and analytical chemists assuming the place they deserve in science and in the perception of the general public ?

Our ambition must be to improve the image of analytical chemistry and to create awareness for its importance both in the public and in other scientific communities. To this end, we also have to continue and to strengthen our efforts in teaching the coming generations of analytical chemists: They not only have to be inducted in the current, fast-evolving repertoire of analytical methods, strategies and instrumentation, but they must also be introduced to a more holistic view of the field, enabling them to more cross-disciplinary interactions instead of the perhaps too often ‘linear’ view of analytical chemistry as a measurement science [4, 32].

Analytical chemists (and their societies) must learn to communicate in a more proactive way. In addition to producing excellent science, analytical chemists must also be good communicators — communicators that address both the scientific community and the general public. The perception of analytical chemistry no longer is that of the ‘kitchenmaid of science’ [33], as it may have appeared to the late H. Malissa sen. back in 1987, but it is to be seen now as that all-embracing information science whose ‘future […] can be found in cross-disciplinary and interdisciplinary areas, focused on solving important societal challenges and engaging young and bright scientists’ [34].

Moreover, we proactively need to raise awareness for the importance of analytical chemistry, analytical tools and the results produced by these for science and society. We need to claim the credits for the vast amount of ingenious work, leading to analytical tools — methods, protocols and reagents — that goes largely unnoticed in the public.

How else could it be explained that the analytical tests for the COVID-19 virus that are performed millions of times every day (PCR and antigen tests) and that allow us diagnose and eventually resume control over this disease are not associated with analytical chemistry but the credits are rather given to medicine and biochemistry?

One could say that the whole world is gaining interest in (bio-)analytical chemistry without realising. This window of opportunity must now be used! Analysts and their (analytical) chemical societies should now point out the central role of analytical chemistry — from basic research to the above quoted applications in the fight against the pandemic which are of so vital importance to allow a return to normal life — and they shall do this in a media-effective manner. Let us thus call ‘analytics in front of the curtain’ — not only to (further) increase the awareness for the crucial role of analytical chemistry, but also to inform and inspire the public, our sponsors and, even equally important, the next generation of scientists for this central subject. Particularly the events of the last year have impressively and sadly demonstrated that ‘you can only manage what you can measure’ — a quote often attributed to the father of Total Quality Management (TQM), W. Edwards Deming [35]. Or, to close with the words of Lord Kelvin (1824–1907), ‘what you are speaking about, and express it in numbers, you know something about it, when you cannot express it in numbers, your knowledge is of a meagre and unsatisfactory kind’.

## References

[1] Ihde AJ (1961). The Karlsruhe Congress: a centennial retrospective. J Chem Educ.

[2] Mönnich MW (2010). Thriving for unity in chemistry: the first international gathering of chemists. Chem Intern.

[3] Weyer J. Analytische Chemie. In: Geschichte der Chemie Band 2 – 19. und 20. Jahrhundert. Springer Spektrum, Berlin. Heidelberg. 2018. 10.1007/978-3-662-55802-7_12.

[4] Adams F, Adriaens M (2020). The metamorphosis of analytical chemistry. Anal Bioanal Chem.

[5] The Nobel Prize Organisation: All Nobel Prizes in Chemistry. https://www.nobelprize.org/prizes/lists/all-nobel-prizes-in-chemistry/. Accessed 04.08.2023.

[6] Ostwald W. The scientific foundations of analytical chemistry, treated in an elementary manner (translated by McGowan G), 2nd edition, Macmillan, London/New York. 1900. Available online at: https://archive.org/details/foundationsscien00ostwrich/mode/2up. Accessed 04.08.2023.

[7] Paull B (2012). Analytical science – a complex and diverse union. Anal Methods.

[8] Steiner D, Krska R, Malachova A, Taschl I, Sulyok M (2020). Evaluation of matrix effects and extraction efficiencies of LC-MS/MS methods as the essential part for proper validation of multiclass contaminants in complex feed. J Agr Food Chem..

[9] Kresse M, Drinda H, Romanotto A, Speer K (2019). Simultaneous determination of pesticides, mycotoxins, and metabolites as well as other contaminants in cereals by LC × LC-MS/MS. J Chromatogr B.

[10] Malachová A, Stránská M, Václavíková M, Elliott CT, Black C, Meneely J, Hajšlová J, Ezekiel CN, Schuhmacher R, Krska R (2018). Advanced LC–MS-based methods to study the co-occurrence and metabolization of multiple mycotoxins in cereals and cereal-based food. Anal Bioanal Chem.

[11] Ferrer I, Writer JH, Keen OS, Lester Y, Padilla-Sánchez JA, Fernández-Ramos C, Thurman EM. LC-TOF-MS for the identification of environmental metabolites and degradation products. Chapter 8 in: Comprehensive analytical chemistry (edited by Pérez S, Eichhorn P, Barceló D) 71: 231–261. Elsevier, Amsterdam. 2016. 10.1016/bs.coac.2016.01.005.

[12] Pérez S, Eichhorn P, Barceló D (eds.). Applications of time-of-flight and Orbitrap mass spectrometry in environmental, food, doping, and forensic analysis. Comprehensive Analytical Chemistry vol. 71. Elsevier, Amsterdam, 2016.

[13] Hird SJ, Lau BPY, Schuhmacher R, Krska R (2014). Liquid chromatography-mass spectrometry for the determination of chemical contaminants in food. TrAC-Trends Anal Chem..

[14] Krska R (2021). Analytik vor den Vorhang. Nachr Chem.

[15] Valcárcel M (2016). Quo vadis, analytical chemistry?. Anal Bioanal Chem..

[16] Waters Acquity QDa Mass Detector, Waters website: https://www.waters.com/waters/en_US/ACQUITY-QDa-Mass-Detector-for-Chromatographic-Analysis/nav.htm?cid=134761404&locale=en_US. Accessed on 04.08.2023.

[17] Salzer R, Cole-Hamilton D, Hrastelj N, Vilela B (2018). Employment and careers of european chemists (ESEC2). Chem European J.

[18] Ettre LS, Sakodynskii KI (1993). M. S. Tswett and the discovery of chromatography I: Early work (1899–1903). Chromatographia.

[19] Griffiths J (2008). A brief history of mass spectrometry. Anal Chem.

[20] Dyson FJ (2012). Is science mostly driven by ideas or by tools?. Science.

[21] According to the Scopus database. https://www.scopus.com. Accessed 4 Aug 2023.

[22] Lowry OH, Rosebrough NJ, Farr AL, Randall RJ (1951). Protein measurement with the Folin phenol reagent. J Biol Chem.

[23] Hare D, New E. Who are you? Anal Scientist 11/09/2016. Available online: https://theanalyticalscientist.com/fields-applications/who-are-you. Accessed 04.08.2023.

[24] Arthur CL, Pawliszyn J (1990). Solid phase microextraction with thermal desorption using fused silica optical fibers. Anal Chem.

[25] Anastassiades M, Lehotay SJ, Stajnbaher D, Schenck FJ (2003). Fast and easy multiresidue method employing acetonitrile extraction/partitioning and “dispersive solid-phase extraction” for the determination of pesticide residues in produce. J AOAC Intern.

[26] Data taken from the GenScript website: https://www.genscript.com/top-100-most-cited-publications.html (accessed: 04.08.2023).

[27] Lindsey D (1989). Using citation counts as a measure of quality in science measuring what’s measurable rather than what’s valid. Scientometrics.

[28] Van Noorden R, Maher B, Nuzzo R (2014). The top 100 papers. Nature.

[29] Markin P. Alternative measures of scholarly impact are increasingly adopted by funders and publishers. Open Science Resources, July 29, 2017. Available online: https://openscience.com/alternative-measures-of-scholarly-impact-are-increasingly-adopted-by-funders-and-publishers/. Accessed 04.08.2023.

[30] Wang L (2019). GC/MS honored with chemical landmark. Chem & Eng News.

[31] Gustafsson C. Scientific background for the Nobel Prize in chemistry 2020: a tool for genome editing. Kungl. Vetenskaps-Akademien, Stockholm. 2020. Available online: https://www.nobelprize.org/uploads/2020/10/advanced-chemistryprize2020.pdf. Accessed 04.08.2023.

[32] Bergquist J, Emmer Å, Farbrot A, Turner C (2023). Anal Bioanal Chem Research and education in analytical chemistry – industrial and academic perspectives from a survey conducted in Sweden. Anal Bioanal Chem..

[33] Malissa H (1987). Analytical chemistry: kitchenmaid or lady of science — deduction versus induction. Fresenius Z Anal Chem..

[34] Bergquist J, Turner C (2018). Analytical chemistry for a sustainable society – trends and implications. Anal Bioanal Chem..

[35] The W.E. Deming Institute, Myth: if you can’t measure it, you can’t manage it ! https://deming.org/myth-if-you-cant-measure-it-you-cant-manage-it/ (accessed: 04.08.2023).

